# Supplementary material for: Influences of Age, Sex and Smoking Habit on Flavor Recognition in Healthy Population
Source: Int J Environ Res Public Health. 2020 Feb 4;17(3):959. doi: 10.3390/ijerph17030959 (PMC7036887; doi:10.3390/ijerph17030959)
Supplement: Supplementary file 1 [file ijerph-17-00959-s001.zip › Figure_TableS1.pdf]

Supplemental table 1. Percentage of correct recognition for each flavor according to gender and smoking habit.

	Female	Male	Non-smokers	Smokers
Almond	88.8%	89.7%	89.2%	88.0%
Banana	88.4%	86.9%	87.7%	89.2%
Cheese	39.0%	37,4%	35.8%	45.8%
Chocolate	16,9%	15,4%	14.2%	16.9%
Coffee	84.6%	82.2%	83.8%	86.7%
Fish	67.6%	68.2%	67.7%	68.7%
Garlic	62.2%	68.2%	63.8%	62.7%
Green Mint	93.6%	89.7%	91.2%	95.2%
Hazelnut	76.7%	70.1%	78.1%	72.3%
Honey	44.8%	33.6%	43.8%	36.1%
Lemon	75.9%	70.1%	74.6%	72.3%
Licorice	89.2%	85.0%	86.5%	91.6%
Mushroom	72.2%	59.8%	68.1%	68.7%
Mustard	37.3%	44.9%	40.0%	37.3%
Onion	83.8%	71.0%	81.2%	74.7%
Peach	87.1%	84.1%	84.2%	91.6%
Roasted beef	89.2%	82.2%	86.9%	86.7%
Smoked	56.2%	55.1%	70.4%	73.5%
Tea	77.2%	70,1%	70.4%	73.5%
Vanilla	68.1%	60.8%	66.5%	62.7%
Water	70.1%	72.9%	55.8%	57.8%
