# Supplementary material for: Influences of Age, Sex and Smoking Habit on Flavor Recognition in Healthy Population
Source: Int J Environ Res Public Health. 2020 Feb 4;17(3):959. doi: 10.3390/ijerph17030959 (PMC7036887; doi:10.3390/ijerph17030959)
Supplement: Supplementary file 1 [file ijerph-17-00959-s001.zip › Figure_S3.pdf]

Supplementary figure 3

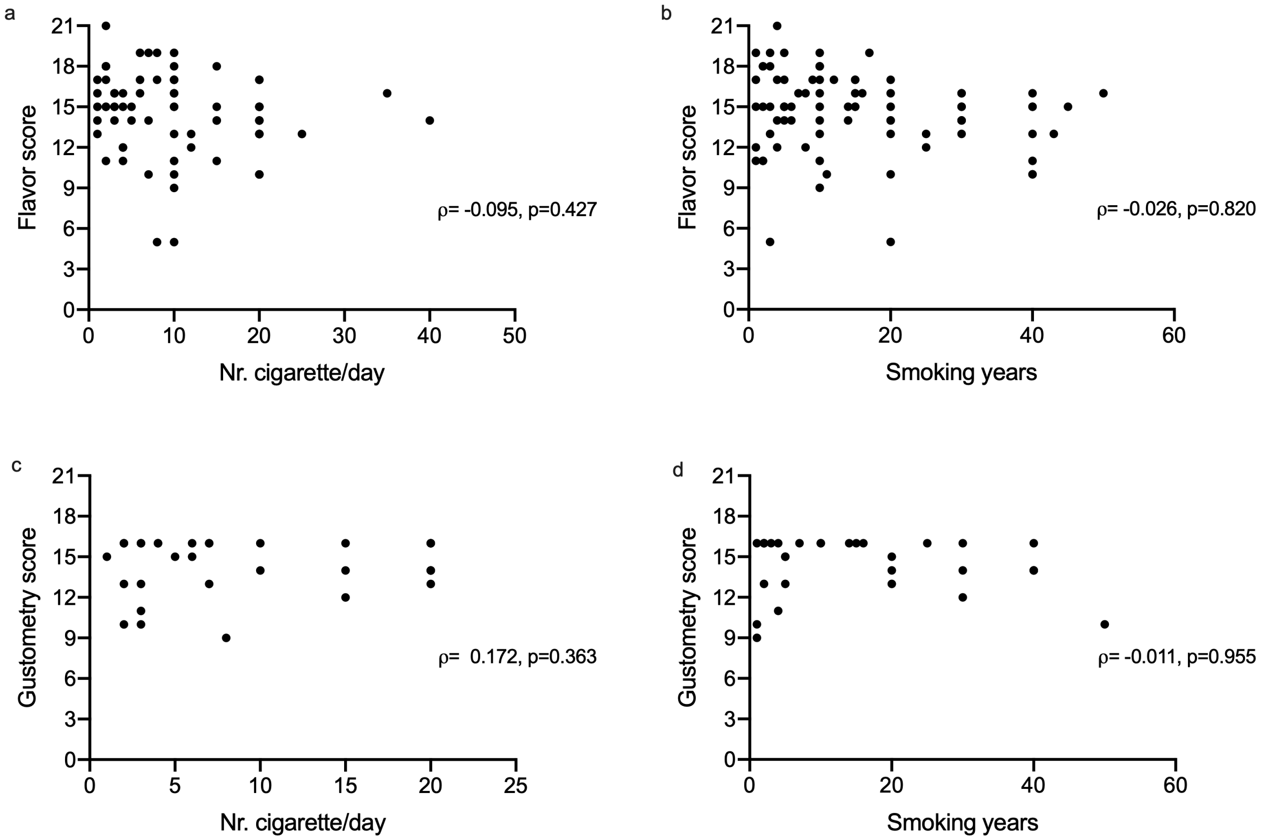

Supplementary figure 3. Flavor and gustometry scores according to the number of smoked cigarette (a, c) and to the cumulative years of smoking (b, d). The number of smoked cigarettes and the cumulated smoked years (corrected for age) did not correlate with either FS or GS.
