# Supplementary material for: Influences of Age, Sex and Smoking Habit on Flavor Recognition in Healthy Population
Source: Int J Environ Res Public Health. 2020 Feb 4;17(3):959. doi: 10.3390/ijerph17030959 (PMC7036887; doi:10.3390/ijerph17030959)
Supplement: Supplementary file 1 [file ijerph-17-00959-s001.zip › Figure_S2.pdf]

Supplementary figure 2

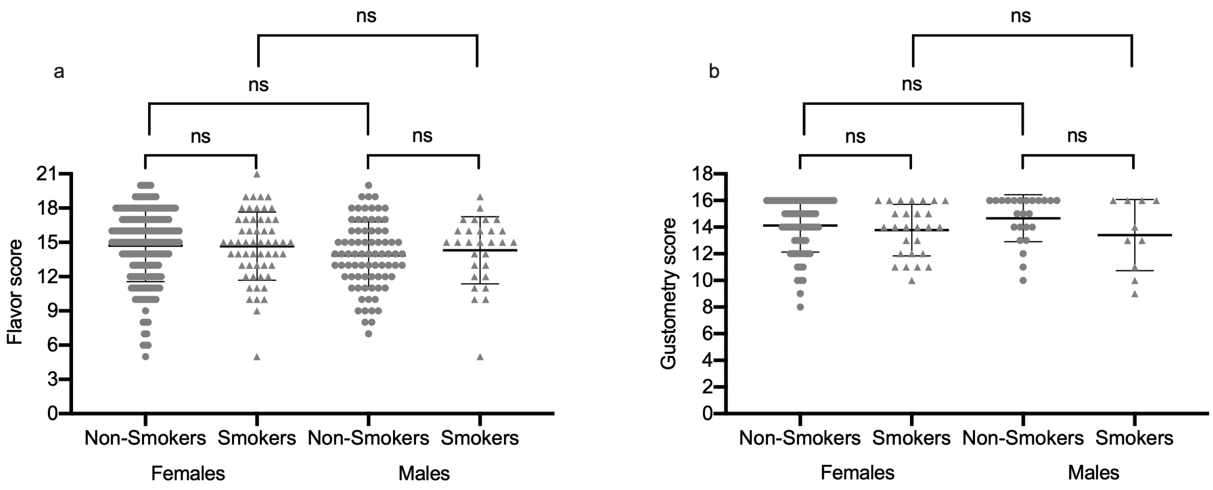

Supplementary figure 2. Flavor (a) and gustometry (b) scores according to the smoke habit and sex. Single values and mean±SD of flavor score for non-smokers and smokers split accordingly to the gender are shown. No significant differences can be determined between smokers and non-smokers belonging to each gender as well as between both females or males with different smoking habit.
