# Supplementary material for: Influences of Age, Sex and Smoking Habit on Flavor Recognition in Healthy Population
Source: Int J Environ Res Public Health. 2020 Feb 4;17(3):959. doi: 10.3390/ijerph17030959 (PMC7036887; doi:10.3390/ijerph17030959)
Supplement: Supplementary file 1 [file ijerph-17-00959-s001.zip › Figure_S5.pdf]

Supplementary figure 5

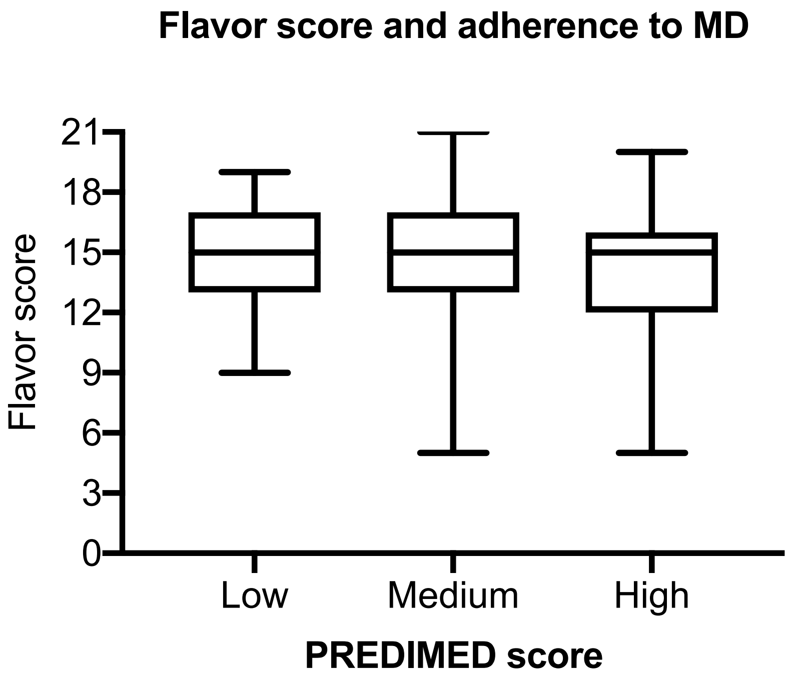

Supplemental Figure 5. Adherence to the Mediterranean diet was evaluated using the previously validated 14-item questionnaire for the assessment of PREvención con DIeta MEDiterránea (PREDIMED) [1] to all the enrolled subjects. PREDIMED adherence to MD was calculated as follows: scores 0–5, low adherence; score 6–9, average adherence; scores ≥10, high adherence. Average of PREDIMED score was 7.99+1.971, median 8 and 5 and 95th percentiles were 5 and 11 respectively. Scores indicating low adherence were present in 8.9%, average adherence was present in 62.9% and high adherence in 19.8% of the studied population.
No correlation was determined between FS and PREDIMED score (not shown), as well as, no differences were determined between adherence classes and FS.

1. Martinez-Gonzalez MA, Salas-Salvado J, Estruch R, Corella D, Fito M, Ros E, Predimed I: **Benefits of the Mediterranean Diet: Insights From the PREDIMED Study.** *Prog Cardiovasc Dis* 2015, **58:**50-60.
