# Supplementary material for: Influences of Age, Sex and Smoking Habit on Flavor Recognition in Healthy Population
Source: Int J Environ Res Public Health. 2020 Feb 4;17(3):959. doi: 10.3390/ijerph17030959 (PMC7036887; doi:10.3390/ijerph17030959)
Supplement: Supplementary file 1 [file ijerph-17-00959-s001.zip › Figure_S4.pdf]

Supplementary figure 4

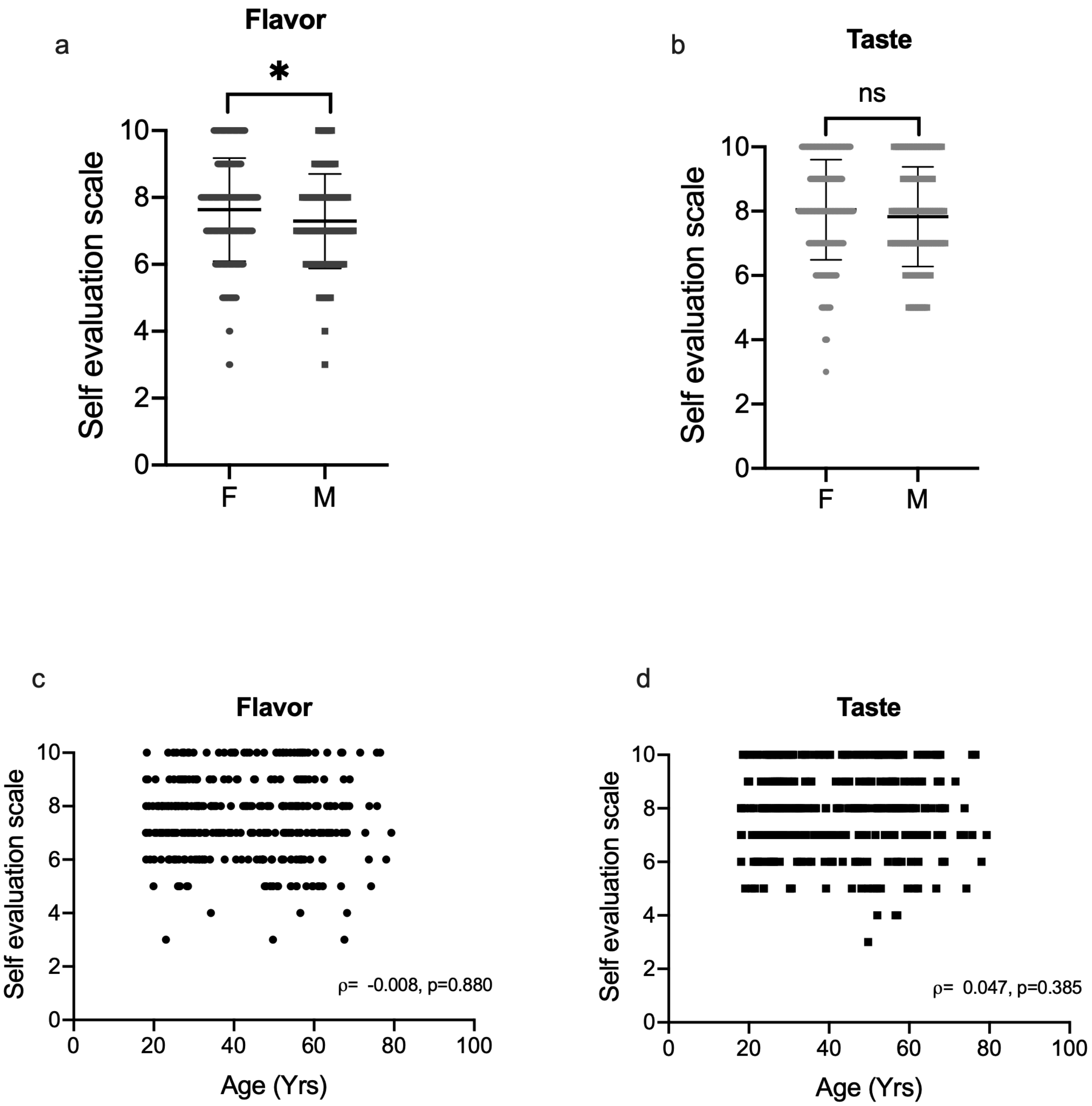

Supplementary figure 4. Results of self-assessment questionnaires. Sex influences on on self-evaluated flavor and taste scores are shown in panel a and b respectively. Panel c shows the correlations between self-evaluated flavor scores and age while in panel d is shown the correlation between self-assessed taste scores and age. * $p < 0.05$, ns = not significant
